# Mindfulness and music performance anxiety among early- and mid-career musicians in transition: the mediating roles of emotional intelligence and perfectionism

**DOI:** 10.3389/fpsyg.2026.1769609

**Published:** 2026-04-15

**Authors:** Haixia Sun, Ziqing Xu, Huilin Wang

**Affiliations:** 1 Li Jinhui Music School, Hunan University of Science and Technology, Xiangtan, China; 2 Business School, Guangdong Ocean University, Yangjiang, China; 3 School of Business, Hunan University of Science and Technology, Xiangtan, China

**Keywords:** emotional intelligence, mindfulness, music performance anxiety, musicians, perfectionism

## Abstract

**Introduction:**

Music performance anxiety is prevalent among professional musicians and can undermine both wellbeing and performance. Musicians navigating the transition from advanced training to professional careers may be particularly vulnerable due to sustained evaluative exposure and career uncertainty. This study examines whether dispositional mindfulness is associated with music performance anxiety and whether this association operates through emotional intelligence and perfectionism, guided by Emotion Regulation Theory.

**Methods:**

Participants were 283 early-career and pre-professional musicians (including advanced conservatory students and working musicians in professional transition) recruited using purposive convenience and snowball sampling. Data were collected via an anonymous online questionnaire. Structural equation modeling (SEM) with AMOS v23 was used to test the hypothesized dual-pathway mediation model. Indirect effects were estimated using bias-corrected bootstrapping (5,000 resamples; 95% confidence intervals).

**Results:**

Dispositional mindfulness was negatively associated with music performance anxiety. Mindfulness was positively associated with emotional intelligence and negatively associated with perfectionism. In turn, emotional intelligence was negatively associated with music performance anxiety, whereas perfectionism was positively associated with music performance anxiety. Bias-corrected bootstrapped confidence intervals indicated a statistically significant indirect association between mindfulness and music performance anxiety through emotional intelligence and perfectionism.

**Discussion:**

The findings are consistent with Emotion Regulation Theory and indicate that mindfulness is statistically associated with lower music performance anxiety via both resource-related (emotional intelligence) and risk-related (perfectionism) pathways. Practically, these results highlight potentially modifiable psychological targets for supporting musicians in early professional development stages, with implications for resilience-building approaches in high-pressure performance communities.

## 1 Introduction

Music performance anxiety refers to the persistent and disruptive emotional-cognitive responses experienced by musicians when facing public performances, evaluative situations, or contexts with high demands for self-presentation (Xu et al., 2025). These responses extend beyond transient “stage fright” and are often accompanied by negative cognitions (e.g., “I will make mistakes”), physiological reactions, and behavioral avoidance (Gómez-Sirvent et al., 2024; Murphy et al., 2025). Previous research has indicated that music performance anxiety not only impairs performance quality-manifested as performance errors, inhibited expression, and reduced creativity (Gómez-López and Sánchez-Cabrero, 2023), but also poses risks to musicians’ mental health, including anxiety, depression, and occupational burnout (Kalenska-Rodzaj, 2023), as well as their career trajectories, such as canceling performances, abandoning musical careers, or transitioning to other professions (Tardif et al., 2024; Barros et al., 2024).

Moreover, musicians navigating the transition from advanced training to professional careers face particularly pronounced challenges. They are often in a period of rapid skill development, frequent public performances, and increasing audience and evaluative pressures, while their professional identities are still being established and career pathways remain uncertain (Latukefu and Pollard, 2022). This transitional stage may include both advanced conservatory students and early-career working musicians, whose exposure to evaluation, competition, and performance demands can be substantial. Consequently, the stress and anxiety experienced by musicians in these career stages tend to be more intense.

In terms of prevalence, existing evidence suggests that music performance anxiety is both widespread and methodologically complex. A systematic review reported prevalence estimates ranging from 16.5 to 60% among professional musicians (Fernholz et al., 2019). However, these estimates were derived from studies that differed substantially in conceptual definitions, measurement instruments, sampling procedures, and diagnostic criteria. In many cases, terms such as music performance anxiety, performance anxiety, and stage fright were used interchangeably, and assessments relied primarily on self-reported symptoms rather than standardized diagnostic evaluations. Consequently, direct comparisons across studies are limited. Despite this heterogeneity, converging evidence indicates that a substantial proportion of musicians experience clinically significant or persistent forms of music performance anxiety. For example, approximately one-third of professional musicians have been reported to suffer from severe and enduring symptoms (Jiang and Tong, 2024). Moreover, recent findings suggest that the overall burden of music performance anxiety has not substantially declined despite increasing awareness and available interventions (Dias et al., 2023).

Taken together, these findings indicate that music performance anxiety extends beyond transient pre-performance nervousness, representing a persistent occupational challenge with considerable conceptual and empirical complexity. This underscores the importance of moving beyond descriptive prevalence data to examine the underlying psychological mechanisms that differentiate adaptive from maladaptive responses. Musicians navigating early professional development and transitional stages constitute a particularly informative and practically relevant group for such research, as they face frequent evaluative pressures and career uncertainty. From a positive psychology perspective, these performance-based musicians are especially vulnerable to sustained anxiety, making them a meaningful target for studies on protective and risk-related psychological traits, as well as resilience-building interventions.

However, the persistence of music performance anxiety despite available interventions suggests that additional long-term, trait-based avenues for psychological support are still needed. Although various interventions for music performance anxiety, such as cognitive-behavioral therapy (Kinney et al., 2025; Martins et al., 2025; Latukefu and Pollard, 2022), exposure therapy (Bellinger et al., 2023), and performance simulation training (Borges et al., 2023), have demonstrated some effectiveness, these approaches primarily focus on short-term state regulation in performance contexts, paying relatively little attention to the psychological traits that support professional musicians over the course of their careers. Against the backdrop of increasing occupational competition, evaluative pressure, and performance exposure, understanding how individual internal resources can enhance resilience and sustain stable performance has become a critical topic of scholarly interest.

Within this context, mindfulness has emerged as a potential protective factor against music performance anxiety (Xiang and Li, 2025). Mindfulness involves accepting and non-judgmental awareness of present-moment experiences, including emotions, thoughts, and bodily sensations. It reduces negative automatic thoughts, self-critical rumination, and excessive self-monitoring, while enhancing emotional regulation and attentional control, capacities crucial for high-pressure performance situations (Lecuona et al., 2023). Empirical evidence suggests that dispositional mindfulness is negatively associated with both performance anxiety and negative affect (Boileau et al., 2024), and short-term mindfulness interventions can effectively reduce pre- and post-performance anxiety in musicians (Paese and Egermann, 2024).

Despite increasing research on mindfulness in music performance, several gaps remain. Most studies focus on student populations, whereas early-career musicians-defined here as individuals transitioning from advanced training to professional practice, including advanced conservatory students and musicians in the first years of professional work-remain underrepresented (Patston and Osborne, 2016; Latukefu and Pollard, 2022). Prior research has largely examined single psychological factors in isolation, such as self-efficacy or emotional states (Kalenska-Rodzaj, 2023), neglecting the multi-pathway mechanisms through which mindfulness may influence anxiety outcomes. In particular, emotional intelligence, a protective capacity, and perfectionism, a vulnerability trait, are both strongly linked to performance anxiety (Egan et al., 2022; Ferber et al., 2024; Zou and Park, 2024), yet few studies have integrated them within a single framework to capture their concurrent effects. Furthermore, although Emotion Regulation Theory provides a theoretical basis for understanding anxiety and coping processes (Lee et al., 2016), its application to music performance anxiety in transitional professional contexts remains limited. The literature also rarely integrates both protective and vulnerability traits in ways that can inform strength-based interventions for musicians with limited access to formal psychological support. To address these gaps, the present study examines early-career musicians and tests a model in which mindfulness influences music performance anxiety through both emotional intelligence and perfectionism as concurrent pathways, allowing for an integrated understanding of how protective and vulnerability traits jointly shape anxiety outcomes in high-pressure performance contexts.

Drawing upon Conservation of Resources theory (Hobfoll, 1989), the present study conceptualizes mindfulness as a psychological resource that helps individuals preserve, mobilize, and replenish emotional and cognitive energy when facing performance-related stressors. Within high-evaluative performance contexts, such as professional music settings, anxiety can be understood as a response to perceived or actual resource loss. From this perspective, mindfulness functions as a foundational personal resource that buffers against resource depletion and supports adaptive coping under sustained evaluative pressure.

While Conservation of Resources theory clarifies why mindfulness may be broadly protective in demanding performance environments, emotion regulation theory (Gross, 1998) helps explain how this protective effect operates at the psychological process level. Mindfulness enhances individuals’ capacity to perceive, understand, and regulate emotional experiences through non-judgmental awareness and attentional stability. These regulatory processes closely align with the core components of emotional intelligence, including emotional awareness, emotional clarity, and effective emotion management. Musicians high in dispositional mindfulness may therefore demonstrate stronger emotional intelligence, enabling them to regulate physiological arousal, reinterpret evaluative cues, and maintain composure during high-stakes performances. At the same time, perfectionism represents a vulnerability-related cognitive-evaluative disposition characterized by excessively high personal standards and heightened concerns over mistakes (Dorsey, 2025). In performance settings, maladaptive perfectionism often intensifies fear of negative evaluation and self-critical rumination, thereby amplifying anxiety responses. From a resource perspective, rigid perfectionistic concerns may accelerate psychological resource depletion by sustaining threat-focused cognition. Mindfulness, through reduced cognitive fusion with evaluative thoughts and greater acceptance of imperfection, may weaken these rigid standards and mitigate their anxiety-provoking effects.

Importantly, emotional intelligence and perfectionism are not conceptually independent traits. Both involve self-evaluative and regulatory processes in emotionally charged performance contexts. Higher emotional intelligence may buffer maladaptive perfectionism by enabling more flexible interpretations of performance standards and more adaptive responses to mistakes (Perrone-McGovern et al., 2017). Conversely, rigid perfectionistic concerns may disrupt effective emotional processing and undermine regulation capacities. These interconnections suggest that adaptive emotional resources and maladaptive evaluative tendencies operate in concert rather than isolation in shaping music performance anxiety. From a Conservation of Resources perspective, emotional intelligence may function as a secondary regulatory resource that protects individuals from resource loss triggered by rigid self-evaluative standards. Musicians with higher emotional intelligence are better able to reinterpret performance setbacks, regulate self-critical emotions, and maintain psychological flexibility, thereby reducing maladaptive perfectionistic concerns. Within the emotion regulation framework, enhanced emotional awareness and reappraisal capacities may weaken the rigid cognitive patterns that characterize maladaptive perfectionism. By simultaneously considering resource-based protection and vulnerability-related cognition, the present study seeks to provide a strength-informed understanding of how mindfulness influences music performance anxiety among musicians in early professional transition.

Thus, this study proposes the following hypothesis:

*H1*: Mindfulness is positively associated with emotional intelligence.

*H2*: Mindfulness is negatively associated with perfectionism.

*H3*: Emotional intelligence is negatively associated with perfectionism.

*H4*: Emotional intelligence is negatively associated with music performance anxiety.

*H5*: Perfectionism is positively associated with music performance anxiety.

*H6*: Emotional intelligence and perfectionism mediate the relationship between mindfulness and music performance anxiety.

**FIGURE 1:**
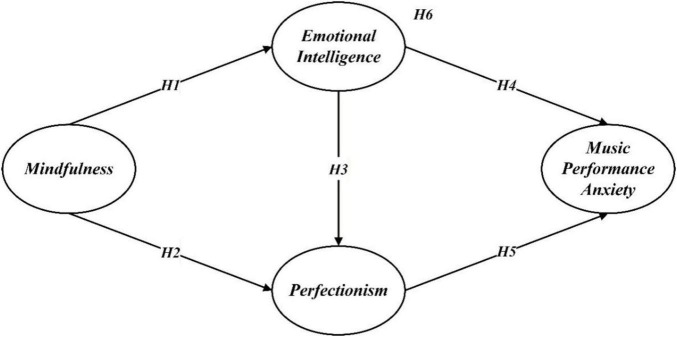
Hypothesis model.

All hypotheses are summarized in Figure 1.

## 2 Materials and methods

### 2.1 Participants and procedures

Participants were recruited in September from universities and conservatories in southern China. Using purposive convenience sampling supplemented by snowballing, invitations were distributed via departmental mailing lists and WeChat communities for musicians (including conservatory- and industry-based groups). The target population comprised early- and mid-career professional and pre-professional musicians.

Eligibility additionally required active engagement in professional music activities (e.g., paid performance, teaching, or contract-based work) and/or ongoing advanced conservatory training, regular practice, and at least one public performance in the past 12 months. Accordingly, the sample included both advanced conservatory students preparing for professional careers and early-career musicians already engaged in professional practice. This operationalization reflects the transitional nature of the early- to mid-career stage rather than a strictly full-time professional classification. Professional status (e.g., student vs. working professional) was not directly collected as a separate demographic variable in the present study. Therefore, the sample should be interpreted as comprising musicians in transition, rather than formally classified subgroups. Data were collected via an anonymous online questionnaire after electronic informed consent. The online survey employed mandatory response settings; therefore, no missing data were observed in the final dataset.

Data collection was paused upon reaching 300 submissions. We then applied pre-specified quality checks and excluded cases for any of the following reasons: (a) straight-lining/identical responses across items, (b) completion time below a conservative threshold based on the sample distribution, or (c) anomalous IP information (duplicates, cross-region, or inconsistent with the stated sampling frame). All exclusions were conducted prior to structural analyses and were based on predefined criteria. The final analytic sample comprised 283 valid cases, yielding a data retention rate of 94.3%.

Sample characteristics are reported in Table 1 (gender, age bands, primary performance type, average daily practice time, public performance experience, and prior psychological/mindfulness training). Because professional status was not collected as a separate variable, the proportion of students versus working professionals could not be reported in Table 1. As shown in Table 1, the sample was relatively balanced in terms of gender, with a slightly higher proportion of female participants than male participants. Most participants were between 23 and 32 years old, reflecting the targeted early- to mid-career stage. In terms of performance specialization, the majority of participants were instrumental musicians, followed by vocal performers, with a smaller proportion engaged in conducting or other performance roles.

**TABLE 1:** Demographic characteristics (*n* = 283).

Profiles	Options	*n* (%)
Gender	Male	128 (45.2)
	Female	155 (54.8)
Age	18–22	34 (12.0)
	23–27	73 (25.8)
	28–32	115 (40.6)
	33 or above	61 (21.6)
Primary performance type	Instrumental	159 (56.2)
	Vocal	91 (32.2)
	Conducting	25 (8.8)
	Other	8 (2.8)
Average daily practice time	Less than 1 h	27 (9.5)
	1–2 h	51 (18.0)
	3–4 h	121 (42.8)
	More than 4 h	84 (29.7)
Experience of public performance	3 times or fewer	27 (9.5)
	4–6 times	126 (44.5)
	7 times or more	130 (45.9)
Have you received psychological or mindfulness training?	Yes	91 (32.2)
	No	192 (67.8)

Regarding practice and performance experience, most participants reported practicing at least 3 h per day, and nearly half had extensive public performance experience, having performed seven times or more in the past year. With respect to psychological background, approximately one-third of the participants reported having received prior psychological or mindfulness-related training, while the remaining participants had not received such training.

Overall, the sample reflects a population of early- and mid-career musicians situated in advanced professional training and/or early stages of professional engagement, with substantial performance exposure and varying levels of psychological training experience, providing an appropriate context for examining music performance anxiety and its psychological mechanisms.

### 2.2 Instruments

We collected basic demographics and background characteristics: gender, age, primary performance type, average daily practice time, public performance experience, and prior exposure to psychological or mindfulness training. These variables were descriptively examined; however, the primary aim of the study was theory-driven model testing rather than demographic prediction. Therefore, demographic variables were not included in the structural model.

Mindfulness was measured with the 5-item Mindful Attention Awareness Scale (MAAS-5) (Van Dam et al., 2010). The MAAS-5 assesses individuals’ tendency to attend to and be aware of present-moment experiences in daily life. Consistent with prior research, the scale captures mindfulness as a trait-like characteristic rather than a situational state. To enhance clarity for the target sample, the original reverse-worded items were linguistically adapted into semantically equivalent positively worded statements. Participants rated each item on a Likert scale, and mean scores were calculated, with higher scores indicating greater mindfulness. Confirmatory factor analysis supported the intended unidimensional structure and indicated satisfactory reliability following this adaptation.

Emotional intelligence was measured with the Brief Emotional Intelligence Scale (BEIS-10) (Davies et al., 2010). This scale measures individuals’ perceived ability to recognize, understand, regulate, and utilize emotions in themselves and others. The BEIS-10 has demonstrated good psychometric properties in diverse populations and is particularly suitable for contexts involving emotional demands and interpersonal evaluation. Sample item includes “I have control over my emotions.” Responses were averaged to create a composite emotional intelligence score, with higher values reflecting higher emotional intelligence.

Perfectionism was measured with the Multidimensional Perfectionism Scale (F-MPS) (Burgess et al., 2016). Although the F-MPS comprises multiple subdimensions, the present study focused on overall perfectionistic self-evaluative tendencies relevant to performance contexts and therefore used a composite score. In high-pressure performance environments, rigid self-standards and heightened concern over mistakes often operate jointly as a generalized self-evaluative pattern. Accordingly, the aggregated score was used to capture this broader evaluative orientation, while acknowledging that adaptive and maladaptive dimensions may function differently in other contexts.

Music performance anxiety was assessed with the Short Performance Anxiety Scale for Musicians (Mazzarolo and Schubert, 2022). This scale captures key dimensions of performance anxiety, including emotional arousal, cognitive concern, avoidance tendencies, and negative affect associated with musical performance. Sample items include “I experience strong nerves or anxiety before I perform.” Participants rated each item based on their typical performance experiences, and mean scores were computed, with higher scores indicating greater levels of music performance anxiety.

### 2.3 Data analysis

Data were analyzed using covariance-based SEM in AMOS v23. We ran a confirmatory factor analysis (CFA) to assess reliability (Cronbach’s α, CR) and validity (loadings, AVE, Fornell–Larcker), then estimated the structural model to test hypotheses. Indirect effects were estimated using bias-corrected percentile bootstrapping with 5,000 resamples and 95% confidence intervals. Model fit was evaluated using χ^2^/df, NFI, IFI, TLI, CFI, and RMSEA. All structural paths were specified as linear relationships.

Researchers addressed potential common method variance (CMV) from self-reports by following Mossholder et al. (1998) procedure. Specifically, we compared a single-factor CMV model with the hypothesized multi-factor measurement model using χ^2^ and degrees of freedom. The single-factor model fit the data poorly [χ^2^(377) = 7501.799, *p* < 0.001] relative to the multi-factor model [χ^2^(344) = 1096.756, *p* < 0.001], yielding a substantial difference [Δχ^2^(33) = 6405.043, *p* < 0.001]. While this result suggests that a single common factor does not fully account for the covariance among the measures, this approach represents a necessary but not sufficient test of CMV. Accordingly, CMV cannot be entirely ruled out and should be considered when interpreting the results.

## 3 Results

### 3.1 Measurement model

Reliability and validity were evaluated using CFA in AMOS v23. All constructs exhibited excellent internal consistency, with Cronbach’s α values exceeding 0.90 (Table 2), consistent with Fornell and Larcker (1981). Convergent validity was supported by AVE values above 0.60 (exceeding the 0.50 benchmark) and composite reliability (CR) values above 0.80 for every construct, alongside strong standardized factor loadings ranging from 0.788 to 0.907 (Table 2). Discriminant validity was also demonstrated, as the square roots of the AVE on the diagonal surpassed the corresponding inter-construct correlations (Table 3). Collectively, these results indicate that the measurement model possesses robust reliability as well as convergent and discriminant validity.

**TABLE 2:** Reliability and validity.

Items	Loadings	Cronbach’s α	CR	AVE
Mindfulness (MIN)		0.941	0.942	0.764
MIN1	0.891			
MIN2	0.899			
MIN3	0.865			
MIN4	0.883			
MIN5	0.831			
Emotional intelligence (EI)		0.963	0.964	0.726
EI1	0.841			
EI2	0.854			
EI3	0.895			
EI4	0.870			
EI5	0.831			
EI6	0.809			
EI7	0.825			
EI8	0.871			
EI9	0.868			
EI10	0.851			
Perfectionism (PER)		0.944	0.944	0.679
PER1	0.842			
PER2	0.850			
PER3	0.838			
PER4	0.788			
PER5	0.834			
PER6	0.829			
PER7	0.805			
PER8	0.804			
Music performance anxiety (MPA)		0.937	0.937	0.747
MPA1	0.907			
MPA2	0.897			
MPA3	0.833			
MPA4	0.863			
MPA5	0.818			

**TABLE 3:** Pearson correlation.

Construct	MIN	EI	PER	MPA
MIN	(0.874)	(0.852)	(0.824)	(0.864)
EI	0.556 **			
PER	−0.450 **	−0.376 **		
MPA	−0.303 **	−0.502 **	0.548 **	

Values on the diagonal represent the square root of AVE, while off-diagonals indicate Pearson correlations between constructs. **p < 0.01.

To further examine the adapted MAAS-5, a separate single-factor CFA was conducted. The model showed an acceptable fit to the data, χ^2^/df = 2.874, CFI = 0.993, TLI = 0.985, IFI = 0.993, GFI = 0.979, and RMSEA = 0.082. Standardized factor loadings ranged from 0.831 to 0.899. These results support the unidimensional structure of the adapted MAAS-5.

### 3.2 Structural model

**FIGURE 2:**
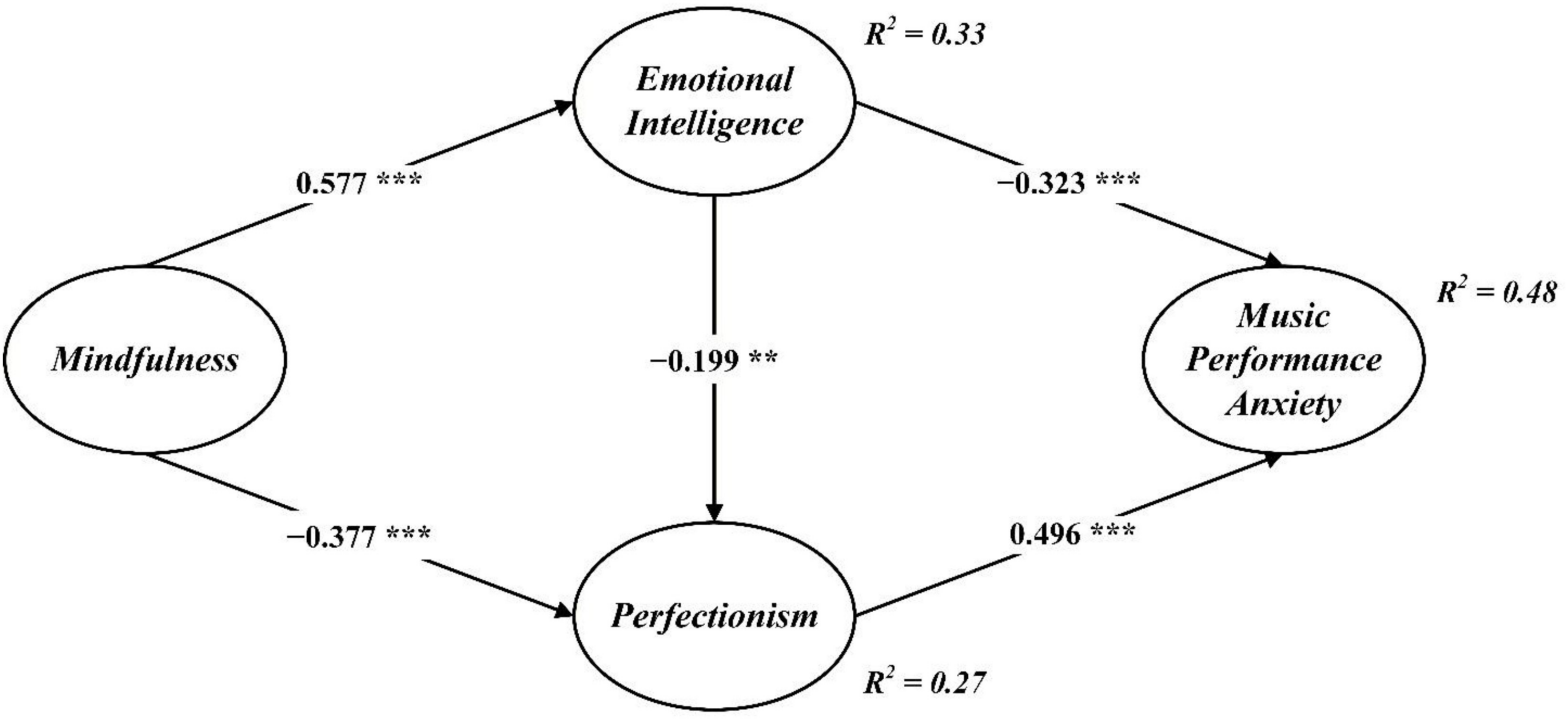
Structural model. **p < 0.01, ***p < 0.001.

After establishing sound measurement properties, we estimated the structural model in AMOS v23 to test the hypotheses. Model fit indices were as follows: χ^2^/df = 2.339, NFI = 0.905, IFI = 0.943, TLI = 0.936, CFI = 0.943, RMSEA = 0.069. These values fall within commonly accepted ranges and suggest that the model provides an adequate representation of the observed data. Zero-order associations among the constructs are reported in Table 2. Standardized path coefficients are shown in Figure 2.

As depicted in Figure 2, mindfulness was positively associated with emotional intelligence (β = 0.577, *p* < 0.001) and negatively associated with perfectionism (β = −0.377, *p* < 0.001), supporting H1 and H2. Emotional intelligence was negatively related to perfectionism (β = −0.199, *p* < 0.01; H3) and to music performance anxiety (β = −0.323, *p* < 0.001; H4). Perfectionism, in turn, was positively related to music performance anxiety (β = 0.496, *p* < 0.001; H5).

Mediation was evaluated using bias-corrected bootstrapping (5,000 resamples; 95% CIs). As summarized in Table 4, the results indicate that mindfulness exerts significant indirect effects on music performance anxiety through both emotional intelligence and perfectionism. Specifically, the indirect effect via emotional intelligence was −0.227 [95% CI (−0.323, −0.151), *p* < 0.001], and the indirect effect via perfectionism was −0.209 [95% CI (−0.336, −0.113), *p* < 0.001]. In addition, the sequential pathway from mindfulness → emotional intelligence → perfectionism → music performance anxiety was also significant [−0.061, 95% CI (−0.130, −0.016), *p* = 0.009]. The total indirect effect combining all mediating pathways was −0.497, indicating that nearly half of the total effect of mindfulness on music performance anxiety is transmitted via these mediators. These findings provide robust support of H6.

**TABLE 4:** Standardized indirect effect.

Path	Point estimate	Bootstrapping		
		Bias-corrected 95% CI		Two-tailed significance
		Lower	Upper	
MIN → MPA	−0.347	−0.472	−0.228	0.000
MIN → EI → MPA	−0.227	−0.323	−0.151	0.000
MIN → PER → MPA	−0.209	−0.336	−0.113	0.000
MIN → EI → PER → MPA	−0.061	−0.130	−0.016	0.009

## 4 Discussion

Early- and mid-career professional musicians, represent a performance-based occupational group exposed to sustained evaluation, career uncertainty, and elevated anxiety risk. The present findings help clarify modifiable psychological pathways that may support resilience and wellbeing in high-pressure performance communities.

### 4.1 Theoretical contributions

This study makes several important theoretical contributions to the literature on mindfulness, emotion regulation, and music performance anxiety.

First, this research provides empirical support for the applicability of Emotion Regulation Theory within a sustained high-pressure professional performance context. Although Emotion Regulation Theory has been widely applied to clinical, educational, and general occupational settings, its application to professional music performance has received relatively limited attention. The present study conceptualizes early- and mid-career musicians as occupying a transitional stage. Musicians in this stage are repeatedly exposed to evaluative scrutiny, public judgment, and performance-contingent outcomes, making their emotional experiences distinct from episodic stress contexts. The finding that dispositional mindfulness is associated with lower music performance anxiety among early- and mid-career professional musicians is consistent with the view that emotion regulation processes remain relevant in sustained, career-relevant performance environments. In this way, the study situates music performance anxiety within broader emotion regulation research rather than treating it as a domain-specific exception.

Second, this study offers an integrated examination of the mechanisms linking mindfulness to music performance anxiety by incorporating emotional intelligence and perfectionism within a unified dual-pathway framework. While prior research has explored various mediating processes separately, the present study evaluates adaptive and maladaptive mechanisms simultaneously within the same structural model. Emotional intelligence represents individuals’ capacity to perceive, understand, and manage emotions (Salovey and Grewal, 2005), reflecting a resource-building process aligned with effective emotion regulation. In contrast, perfectionism reflects rigid self-evaluative standards and heightened fear of mistakes (Lo and Abbott, 2013), representing a cognitive vulnerability that may intensify anxiety in evaluative contexts. By examining these two constructs concurrently, the findings suggest that mindfulness is associated with lower music performance anxiety through both the cultivation of emotional competencies and the attenuation of maladaptive self-evaluative tendencies. This integrated perspective contributes to a more differentiated understanding of emotion regulation processes in performance settings, highlighting the importance of considering both resource-enhancing and risk-reducing pathways. At the same time, emotional intelligence and perfectionism may not function as entirely independent processes. Their potential conceptual overlap, reciprocal influence, or sequential relationship warrants further investigation in future longitudinal research.

Third, this study contributes to the theoretical understanding of mindfulness by positioning dispositional mindfulness as a relatively stable self-regulatory capacity relevant to professional functioning. Whereas much prior research has focused on short-term mindfulness-based interventions and their immediate effects (Zhang et al., 2021; Howarth et al., 2019), the present findings highlight the relevance of naturally occurring individual differences in mindfulness within a sustained performance context. Rather than conceptualizing mindfulness solely as a transient attentional state, the results suggest that it may function as a foundational regulatory orientation that shapes how individuals engage with emotional experiences and evaluative demands over time. In this sense, dispositional mindfulness appears to be associated with broader patterns of emotional functioning, including higher emotional intelligence and lower perfectionistic concerns. This perspective aligns with theoretical views that regard mindfulness as a core psychological resource that may support adaptive functioning in high-pressure environments, while inviting future research to further clarify its developmental and longitudinal dynamics.

Finally, by focusing on early- and mid-career professional musicians, this study addresses an important population-level gap in the literature. Much prior research on music performance anxiety has concentrated on student samples or short-term training contexts (Xu et al., 2025), leaving open questions about whether established mechanisms apply to professional musicians navigating ongoing career pressures. The present study contributes by examining individuals situated at the intersection of advanced training and early professional engagement, thereby extending existing findings beyond purely student-based samples while acknowledging the transitional nature of this career stage. By demonstrating that emotion regulation processes grounded in mindfulness remain salient at this career stage, the present study enhances the ecological validity of existing theories and provides a stronger theoretical foundation for understanding music performance anxiety as a persistent, career-related psychological phenomenon.

### 4.2 Practical implications

The findings of this study offer several implications.

First, at the level of findings directly supported by the present data, the results indicate that dispositional mindfulness, emotional intelligence, and perfectionism are statistically associated with music performance anxiety in musicians navigating early professional development. These findings suggest that these constructs represent meaningful psychological correlates of performance anxiety within this population.

Second, as logical extensions, the observed associations imply that mindfulness-related capacities and emotion-regulation competencies may represent potential targets for future intervention research. However, the present study measured dispositional traits rather than intervention effects. Therefore, suggestions such as incorporating mindfulness-based exercises into rehearsal routines or developing emotion-focused pedagogical elements should be understood as theoretically informed extensions rather than evidence-based prescriptions. Their effectiveness remains to be validated through controlled longitudinal or experimental designs.

Similarly, although perfectionism was statistically associated with higher performance anxiety, the present design does not establish that reducing perfectionism would causally decrease anxiety. Accordingly, recommendations concerning mastery-oriented feedback practices or cognitive reframing strategies should be viewed as plausible applied directions grounded in theory, not as empirically tested intervention outcomes within this study.

Third, at a broader contextual level, the findings highlight the potential relevance of psychological resources and cognitive vulnerabilities during transitional career stages. While institutional initiatives (e.g., workshops or structured support programs) may represent reasonable applied considerations, the present data do not directly evaluate such programs. Future research should examine whether structured training efforts produce measurable changes in emotional intelligence, perfectionism, or performance anxiety among musicians in transition.

Finally, the dual-pathway model tested here provides a conceptual framework linking protective and risk-related factors within a single structure. This integrative perspective may inform the design of future multi-component interventions, but causal effectiveness must be established through longitudinal or experimental research before definitive practical recommendations can be made.

### 4.3 Limitations

This study has several limitations that should be explicitly considered when interpreting the findings.

First, a key limitation concerns subgroup classification. Professional status (e.g., advanced conservatory student vs. working musician) was not collected as a separate demographic variable. Consequently, although the sample likely included both advanced conservatory students and early-career musicians, we could not determine their relative proportions, examine whether the observed relationships differed between these subgroups, or assess whether the findings were primarily driven by one subgroup. This limitation should be considered when interpreting the generalizability of the findings.

Second, the cross-sectional design precludes causal inference. Although the dual-pathway model is theoretically grounded and statistically supported, the identified mediation pathways should be interpreted as associative rather than causal mechanisms. The temporal ordering among mindfulness, emotional intelligence, perfectionism, and performance anxiety cannot be established.

Third, all variables were measured using self-report instruments collected at a single time point, raising concerns regarding common method variance (CMV). While a single-factor comparison suggested that a common factor did not fully account for the covariance structure, this test is not sufficient to rule out shared method bias. Simultaneous self-report measurement of dispositional traits and anxiety outcomes may inflate associations. Future research employing longitudinal, multi-source, or experimental designs would strengthen confidence in the robustness of these findings. Additionally, the present analyses assumed linear associations and did not include formal sensitivity or non-linear tests.

Fourth, perfectionism was examined as an overall construct rather than as distinct dimensions. Although this approach was theoretically appropriate for the present study, it may have masked potentially different roles of adaptive and maladaptive perfectionism. Future research should therefore examine dimensional effects more explicitly, as different aspects of perfectionism may operate through different mechanisms in relation to music performance anxiety.

## 5 Conclusion

This study contributes to the understanding of music performance anxiety by examining the role of dispositional mindfulness and its psychological mechanisms among early- to mid-career professional musicians. Consistent with the operationalization adopted in this study, this group includes individuals in advanced professional training as well as those in early stages of professional engagement. Guided by Emotion Regulation Theory, the research demonstrates that mindfulness functions as a trait-like resource that enhances emotional intelligence and mitigates perfectionism, thereby being associated with lower performance-related anxiety.

The findings underscore the importance of considering both protective and risk-related psychological factors in understanding music performance anxiety, moving beyond single-variable explanations to a more comprehensive, dual-pathway perspective. By integrating dispositional mindfulness, emotional intelligence, and perfectionism within a single theoretical framework, this study extends the application of Emotion Regulation Theory to sustained, high-pressure performance contexts and offers theoretically informed insights for interventions aimed at supporting musicians’ psychological wellbeing.

Overall, the results highlight the potential of cultivating mindfulness and adaptive emotion regulation skills as a sustainable approach to alleviating music performance anxiety, while acknowledging that causal effectiveness requires further longitudinal and experimental validation. Taken together, the findings identify modifiable resources and risk processes that may help support resilience in high-pressure performance occupations and transitional professional stages.

## Data Availability

The raw data supporting the conclusions of this article will be made available by the authors, without undue reservation.
